# Integrative network analysis highlights biological processes underlying GLP-1 stimulated insulin secretion: A DIRECT study

**DOI:** 10.1371/journal.pone.0189886

**Published:** 2018-01-02

**Authors:** Valborg Gudmundsdottir, Helle Krogh Pedersen, Karla Viviani Allebrandt, Caroline Brorsson, Nienke van Leeuwen, Karina Banasik, Anubha Mahajan, Christopher J. Groves, Martijn van de Bunt, Adem Y. Dawed, Andreas Fritsche, Harald Staiger, Annemarie M. C. Simonis-Bik, Joris Deelen, Mark H. H. Kramer, Axel Dietrich, Thomas Hübschle, Gonneke Willemsen, Hans-Ulrich Häring, Eco J. C. de Geus, Dorret I. Boomsma, Elisabeth M. W. Eekhoff, Jorge Ferrer, Mark I. McCarthy, Ewan R. Pearson, Ramneek Gupta, Søren Brunak, Leen M. ‘t Hart

**Affiliations:** 1 Department of Bio and Health Informatics, Technical University of Denmark, Kongens Lyngby, Denmark; 2 Department of Translational Bioinformatics, R&D Operations, Sanofi-Aventis Deutschland GmbH, Industriepark Höchst, Frankfurt, Germany; 3 Department of Molecular Cell Biology, Leiden University Medical Center, Leiden, The Netherlands; 4 Novo Nordisk Foundation Center for Protein Research, Disease Systems Biology, Faculty of Health and Medical Sciences, University of Copenhagen, Copenhagen, Denmark; 5 Wellcome Trust Center for Human Genetics, University of Oxford, Oxford, United Kingdom; 6 Oxford NIHR Biomedical Research Center, Oxford, United Kingdom; 7 Oxford Center for Diabetes Endocrinology and Metabolism, University of Oxford, Oxford, United Kingdom; 8 Division of Molecular & Clinical Medicine, School of Medicine, University of Dundee, Dundee, United Kingdom; 9 Department of Internal Medicine, Division of Endocrinology, Diabetology, Angiology, Nephrology and Clinical Chemistry, Eberhard Karls University Tübingen, Member of the German Centre for Diabetes Research (DZD), Tübingen, Germany; 10 Institute of Pharmaceutical Sciences, Department of Pharmacy and Biochemistry, Eberhard Karls University, Tübingen, Germany; 11 Department of Internal Medicine, Diabetes Center and Endocrinology, VU University Medical Center, Amsterdam, The Netherlands; 12 Section Molecular Epidemiology, Leiden University Medical Center, Leiden, The Netherlands; 13 Max Planck Institute for Biology of Ageing, Cologne, Germany; 14 Department GI Endocrinology, R&D Diabetes Division, Sanofi-Aventis Deutschland GmbH, Industriepark Höchst, Frankfurt, Germany; 15 Department of Biological Psychology, Vrije Universiteit and the EMGO Institute for Health and Care Research, VU University Medical Center, Amsterdam, The Netherlands; 16 Netherlands Consortium for Healthy Aging, Leiden, The Netherlands; 17 Section of Epigenomics and Disease, Department of Medicine, Imperial College London, London, United Kingdom; 18 Genomic Programming of Beta Cells Laboratory, Institut d'Investigacions Biomediques August Pi I Sunyer (IDIBAPS), Barcelona, Spain; 19 Centro de Investigación Biomédica en Red de Diabetes y Enfermedades Metabólicas Asociadas (CIBERDEM), Madrid, Spain; NIDCR/NIH, UNITED STATES

## Abstract

Glucagon-like peptide 1 (GLP-1) stimulated insulin secretion has a considerable heritable component as estimated from twin studies, yet few genetic variants influencing this phenotype have been identified. We performed the first genome-wide association study (GWAS) of GLP-1 stimulated insulin secretion in non-diabetic individuals from the Netherlands Twin register (n = 126). This GWAS was enhanced using a tissue-specific protein-protein interaction network approach. We identified a beta-cell protein-protein interaction module that was significantly enriched for low gene scores based on the GWAS *P*-values and found support at the network level in an independent cohort from Tübingen, Germany (n = 100). Additionally, a polygenic risk score based on SNPs prioritized from the network was associated (*P* < 0.05) with glucose-stimulated insulin secretion phenotypes in up to 5,318 individuals in MAGIC cohorts. The network contains both known and novel genes in the context of insulin secretion and is enriched for members of the focal adhesion, extracellular-matrix receptor interaction, actin cytoskeleton regulation, Rap1 and PI3K-Akt signaling pathways. Adipose tissue is, like the beta-cell, one of the target tissues of GLP-1 and we thus hypothesized that similar networks might be functional in both tissues. In order to verify peripheral effects of GLP-1 stimulation, we compared the transcriptome profiling of ob/ob mice treated with liraglutide, a clinically used GLP-1 receptor agonist, versus baseline controls. Some of the upstream regulators of differentially expressed genes in the white adipose tissue of ob/ob mice were also detected in the human beta-cell network of genes associated with GLP-1 stimulated insulin secretion. The findings provide biological insight into the mechanisms through which the effects of GLP-1 may be modulated and highlight a potential role of the beta-cell expressed genes *RYR2*, *GDI2*, *KIAA0232*, *COL4A1* and *COL4A2* in GLP-1 stimulated insulin secretion.

## Introduction

Glucagon-like petide-1 (GLP-1) receptor agonists and DPP4-inhibitors are increasingly used therapeutic agents for type 2 diabetes, as they stimulate insulin secretion from the pancreatic beta-cells by potentiating glucose-dependent insulin secretion. In addition to the effects on the pancreas these drugs also operate via effects on other tissues. For instance, liraglutide, a clinically used GLP-1 receptor agonist, was shown to have beneficial effects on cardiovascular outcome and body weight loss [1]. However, the response to these drugs varies considerably between individuals. A large part of this variability is expected to be explained by underlying genetic differences as GLP-1 stimulated insulin secretion has an estimated heritability of 0.53 (95% CI, 0.33–0.70) [2]. Identification of these genetic determinants may aid patient stratification with regard to treatment response and shed light on the differential properties of the complex signaling networks controlling GLP-1 stimulated insulin secretion, which to date are not well understood. Previous studies have used targeted genotyping approaches to identify variants associated with GLP-1 stimulated insulin secretion, which mostly focused on GWAS loci for type 2 diabetes or related traits. Among the loci nominally associated with GLP-1 stimulated insulin secretion are variants in the *TCF7L2* [3], *GLP1R* [4], *WFS1* [5] and *CTRB1/2* loci [6] (all *P* < 0.05), which highlights the potential of further genetic studies of GLP-1 stimulated insulin secretion.

Genome-wide association studies (GWAS) have successfully been used to identify genetic variants underlying complex phenotypes but for disease case-control status require large sample sizes to reveal variants with modest or small effect sizes. However, the use of more proximal phenotypes may reduce sample size requirements and furthermore, such analyses can be enhanced using integrative network approaches [7], by integrating genetic information with complementary data types such as tissue-specific gene expression and protein-protein interaction (PPI) data [8,9]. The aim of the current study was to provide insight into the biological mechanisms underlying GLP-1 stimulated insulin secretion using an untargeted integrative genomics approach. We performed a GWAS on 126 nondiabetic individuals from the Netherlands Twin Register (NTR) who underwent a hyperglycemic clamp [10], and the association analysis was augmented with a beta-cell specific PPI network analysis. We identified a set of genes that contained variants associated with GLP-1 stimulated insulin secretion, which at the same time have the potential to physically interact in the beta-cell and are enriched for pathways important for insulin secretion. We carried out validation studies to assess the importance of the prioritized GLP-1 response subnetwork through associations with: i) GLP-1 stimulated insulin secretion in an independent collection of 100 unrelated individuals from Tübingen, Germany[10], ii) glucose stimulated insulin secretion phenotypes in up to 5,318 individuals from MAGIC [11] and iii) gene expression alterations in white adipose tissue as a response to liraglutide (a GLP-1 receptor agonist) treatment in ob/ob mice (a mouse model of obesity).

## Results

### GWAS and PPI network analysis

**Fig 1:**
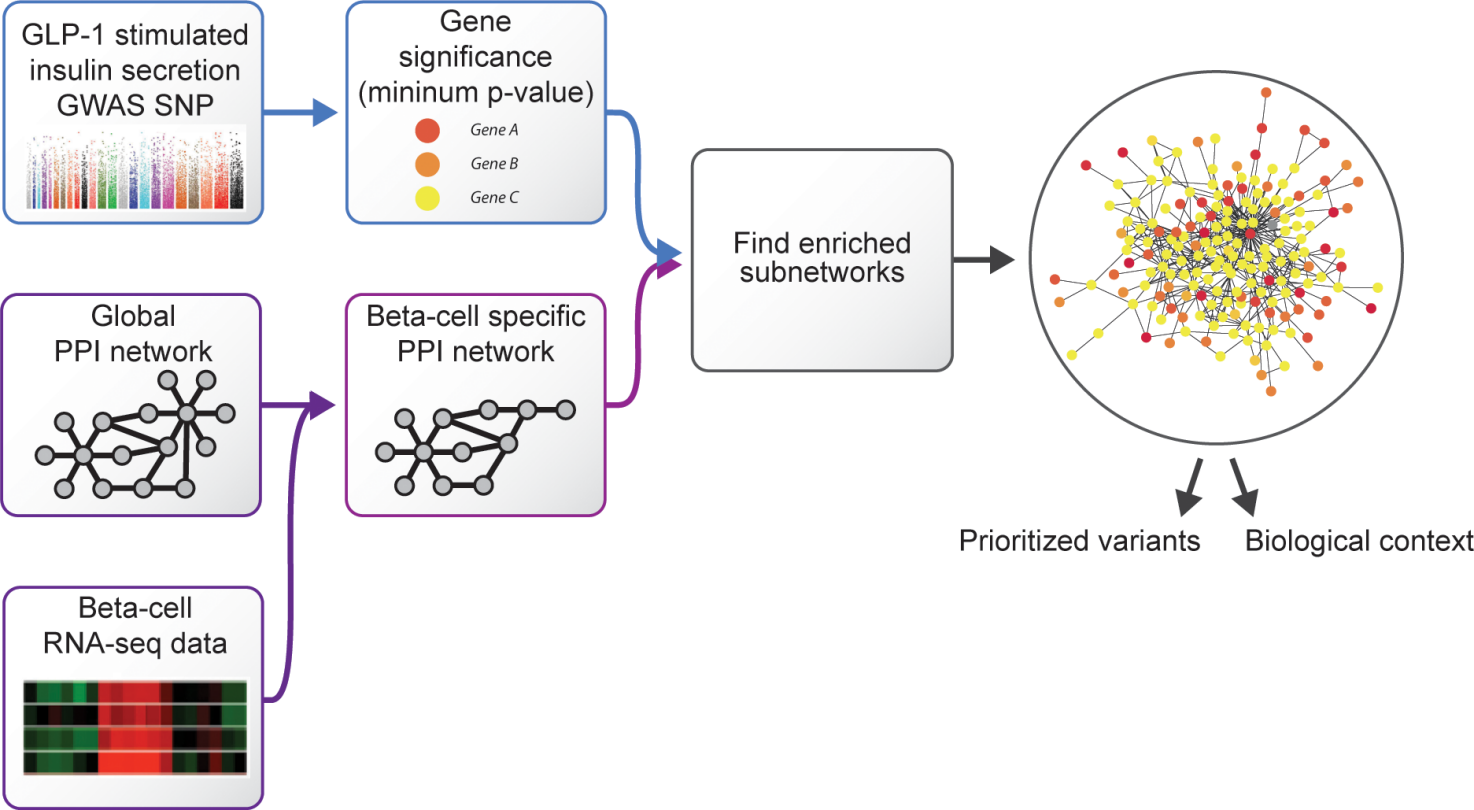
Integrative network analysis workflow overview. GLP-1 stimulated insulin secretion GWAS SNP P-values were converted to gene significance scores, which were then mapped onto a beta-cell specific PPI network created by pruning the global network using beta-cell gene expression data. The jActiveModules algorithm was used to identify network modules that were enriched for association signal. The top scoring network modules were used to prioritize genetic variants and explore the biological context of the genetic associations.

Clinical characteristics for the NTR cohort are shown in Table 1. No single nucleotide polymorphism (SNP) association reached genome-wide significance in the NTR cohort association analysis adjusted for age, gender, BMI, glucose tolerance status and insulin sensitivity (S1 and S2 Figs), while six independent signals were identified with P < 1.0 × 10^−5^ (S1 Table). Using the integrative analysis workflow described in Fig 1, we next sought to identify significant signals at a cellular network-level. A more detailed analysis flowchart is shown in S3 Fig. As tissue-specific PPI networks have previously been shown to perform better for gene prioritization than global networks [12], we mapped gene significance values for GLP-1 stimulated insulin secretion onto a PPI network containing 8,457 genes that are expressed in pancreatic beta-cells [13] (see Methods). We then identified modules in the network that were enriched for genes with the strongest significance values using the jActiveModules algorithm [14] (see Methods for details). The top ranked network module contained 179 genes and had a z-score of 10.11, which was significantly higher than the z-scores of modules obtained by permuted gene scores (S4 Fig).

**Table 1:** Clinical characteristics of the study groups.

	NTR cohort	German cohort
n (NGT/IGT)	120/6	68/32
Age (years)	31.5 ± 6.3	39.7 ± 12.8
Gender (M/F, n)	60/66	44/56
BMI (kg/m^2^)	24.1 ± 3.5	25.8 ± 5.5
Fasting glucose (mmol/l)	4.6 ± 0.4	5.2 ± 0.7
2-hr glucose (mmol/l)	5.4 ± 1.2	6.6 ± 2.1
Fasting insulin (pmol/l)	35 (27–52)	47 (32–67)

Data are means ± SD; median (interquartile range) or number (n).

**Fig 2:**
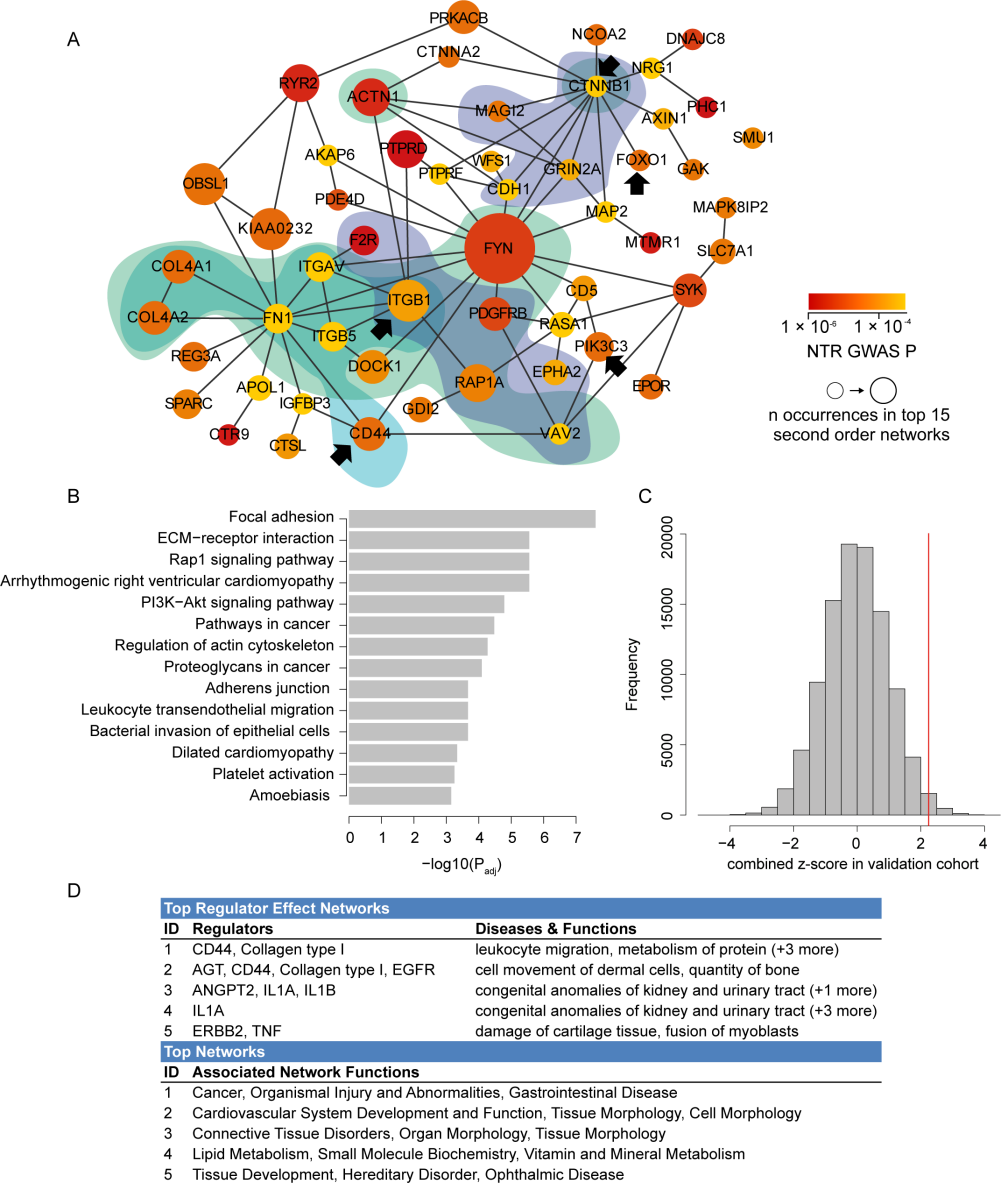
Results from network analysis of GLP-1 stimulated insulin secretion GWAS. A) The beta-cell specific GLP-1 response consensus network, annotated with the top enriched KEGG pathways: Focal adhesion (green), ECM-receptor interaction (blue) and Rap1 signaling (purple). Arrows indicate genes that were identified as upstream regulators of differentially expressed genes in the transcriptome analyses of the liraglutide treated mice versus baseline controls. B) The KEGG pathways enriched (BH adjusted *P*-value < 1 × 10^−3^) within the GLP-1 response consensus network, compared to the whole beta-cell PPI network. C) The red line denotes the combined z-score in the Tübingen validation cohort for 28 consensus network SNPs with discovery GWAS *P* < 5 × 10^−4^ compared to 100,000 z-scores obtained from randomly selected sets of SNPs from the beta-cell network (histogram), empirical *P*-value = 0.012. D) Top panel: Top regulators for networks of differentially expressed genes in the liraglutide treated mice transcriptome experiment. Bottom panel: Prioritized network modules from human and mouse experiments map to connective tissue and focal adhesion related pathways.

To focus on the most important part of this module, we reran the jActiveModules algorithm and created a consensus network from genes occurring in more than one of the top 15 second order modules (see Methods). The resulting consensus network contained 53 genes, whose significance was driven by 51 SNPs (Fig 2A, S2 Table). It contained genes already known to be involved in GLP-1 stimulated insulin secretion (*WFS1* [5], *RYR2* [15], *RAP1A* [16]), glucose stimulated insulin secretion (*VAV2* [17]), mediating the effects of GLP-1 on beta-cell mass (*FOXO1* [18]) and genes implicated in type 2 diabetes through GWAS of Han Chinese [19] and Mexican [20] populations (*PTPRD)*, a gene expression-based genome-wide association study [21] (*CD44*) and a linkage study in an African American population [22], (*MAGI2* and *CTNNA2*).

### Functional investigation of the GLP-1 response consensus network

By definition, all of the genes in the consensus network are expressed in beta-cells, but four (*CTNNA2*, *RYR2*, *GRIN2A*, *NRG1*) have additionally been described as particularly enriched in beta-cells compared to non-beta-cell islet components [13]. The consensus network was significantly enriched for gene ontology (GO) terms related to plasma membrane and signaling transduction (S5 Fig) and enriched KEGG pathways included focal adhesion, extracellular matrix-receptor interaction, the Rap1 and PI3K-Akt signaling pathways and regulation of actin cytoskeleton (Fig 2B).

We investigated if the SNPs driving the significance of the network acted as expression quantitative trait loci (eQTL) for their corresponding genes in the network. We found seven potential eQTL pairs (unadjusted *P* < 0.01) in human islets (n = 118), involving the genes *CTR9*, *RYR2*, *PRKACB*, *DOCK1*, *APOL1*, *ITGB5* and *MAP2* (S3 Table). In addition, we found two potential eQTL pairs for *F2R* and *CD5* in pancreas, and three eQTL pairs in blood samples for *KIAA0232*, *SPARC* and *RAP1A* (S3 Table). We also investigated the overlap of network loci with pancreatic islet regulatory elements and found 15 loci to overlap islet promoter or enhancer regions [23] (S3 Table). Of those, nine loci overlapped clusters of active enhancers, but such clusters are enriched for variants associated with type 2 diabetes and fasting glycemia [23], This was a higher fraction than was observed for the top SNPs from a GWAS performed by MAGIC investigators of corrected insulin response, but lower than for genome-wide significant SNPs for fasting glucose and type 2 diabetes (S6 Fig). Five of the consensus network SNPs (mapping to *APOL1*, *ITGB5*, *MAP2*, *CD5* and *KIAA0232*) reside in loci overlapping active enhancers or clusters of active enhancers in islets and were a part of a potential eQTL pair.

### Validation of the GLP-1 response consensus network

In order to validate the genetic associations driving the network enrichment we calculated a combined z-score for the consensus network SNPs in an independent dataset of 100 unrelated individuals from Tübingen, Germany who underwent a hyperglycemic clamp procedure similar to the NTR cohort (clinical characteristics are shown in Table 1). We restricted all validation attempts to the 31 SNPs (28 of which passed quality control in the Tübingen data) with a discovery GWAS *P* < 5.0 × 10^−4^. The combined z-score based on these 28 SNPs was significantly higher (*P* = 0.01) than those obtained from 100,000 randomly selected sets of SNPs from the beta-cell network (Fig 2C). However, this set of SNPs was not enriched for directional consistency in the validation dataset (14/28 SNPs directionally consistent, *P* = 0.57).

Finally, we investigated if the network analysis prioritization of SNPs has an additional value over the individual SNPs with the lowest *P*-values from the GWAS analysis. We therefore calculated a similar combined z-score from the top 31 ranked independent GWAS SNPs in the NTR discovery cohort but, in contrast to the network-based SNPs, this z-score was not higher in the validation dataset than expected by chance (*P* = 0.95) (S7 Fig).

At the single SNP level, two (rs7669558 and rs72509) of the 31 consensus network SNPs with a discovery GWAS *P* < 5.0 × 10^−4^ had a *P*-value < 0.05 in the validation dataset (S2 Table) but none were significant after Bonferroni correction for multiple testing. We performed a meta-analysis of the discovery and validation dataset (S2 Table), where none of the consensus network SNPs reached a genome-wide significance but four had a meta-analysis *P* < 5.0 × 10^−4^ (*KIAA0232* rs7669558: *P* = 5.9 × 10^−5^, *COL4A1*/*COL4A2* rs72509: *P* = 7.0 × 10^−5^, *RYR2* rs6429033: *P* = 9.4 × 10^−5^ and *GDI2* rs871748: *P* = 1.5 × 10^−4^).

Of the 31 consensus network SNPs, 18 (S3 Table) had available information on both insulin secretion and action indices published by MAGIC (see Methods and S4 Table for detailed overview of phenotypes). Rs871748 (*GDI2*) was nominally associated with four measures of glucose stimulated insulin secretion in the MAGIC data, with a consistent direction of effect compared to the GLP-1 stimulated insulin secretion in the NTR cohort (S5 Table). Furthermore, a weighted polygenic risk score (PRS) made from the 18 SNPs common between the two datasets showed a nominally significant association (*P* < 0.05) with the oral glucose tolerance test derived variables area under the curve (AUC) for insulin, AUC for insulin/AUC for glucose, insulin at 30 minutes and insulin sensitivity index (S6 Table). In contrast, no significant associations were observed when the same number of SNPs was selected based on their discovery GWAS *P*-value alone (S7 Table).

Finally, an independent gene set enrichment analysis was conducted from the transcriptome profiling of white adipose tissue from mice treated with liraglutide (an analogue of GLP-1) versus baseline controls (see S8 Fig and Methods for experimental details). Adipose tissue is, like the beta-cell, one of the target tissues of GLP-1 [24] and we thus hypothesized that similar networks might be functional in both tissues. We identified upstream regulators (see Methods) for the differentially expressed genes (Fig 2D, S8 Table). Interestingly, five upstream regulators predicted to regulate genes in the transcriptome dataset (*CD44*, *FOXO1*, *ITGB1*, *CTNNB1*, and the PI3K complex—which *PIK3CA* is a member of) were also present in the human beta-cell GLP-1 response consensus network (as highlighted in Fig 2A) and the PI3K signaling pathway was furthermore among its top enriched pathways (Fig 2B). Additionally, a member of the collagen Type IV family of genes (*COL4A3*, related to *COL4A1* and *COL4A2*) ranked as a top upstream regulator. *COL4A3* interacts with both *COL4A1* and *COL4A2* and other genes (*ITGAV*, *FN1*, *ITGB1* and *ITGB5*) that appear in the human consensus network, and which connect the collagen pathway to the GLP-1 receptor (S9). *CD44* is predicted to be an upstream regulator of collagen genes and *CTNNB1*, further illustrating how these genes are connected (S9 Fig). Finally, the genes prioritized for GLP-1 response in the human and mouse study were found to be highly connected in tissue-specific functional networks from GIANT [25] for both pancreatic islet and adipose tissue (S10 Fig).

## Discussion

In the present study we describe the first reported GWAS of GLP-1 stimulated insulin secretion. As genetic variants underlying complex phenotypes and diseases are expected to collectively perturb functional modules within the cellular machinery [26], we used the uniquely phenotyped NTR cohort to explore the underlying mechanisms of GLP-1 stimulated insulin secretion using a network analysis approach. While a previous study on the NTR cohort using Metabochip genotyping identified three strong signals for GLP-1 stimulated insulin secretion [6], illustrating the potential for the discovery in this limited sample size, our genome-wide analysis did not reveal any additional loci associated at a genome-wide significance. Instead, we identified a module within a beta-cell specific PPI network that was significantly enriched for gene scores derived from the GWAS. We found support for the network level association in an independent dataset from Germany and a PRS constructed from SNPs selected from the network showed an association with glucose-stimulated insulin secretion phenotypes in the MAGIC consortium. The same support was not observed for a matched number of SNPs selected by the discovery GWAS *P*-value alone, suggesting that the network prioritization approach to some extent enhanced the GWAS findings. While GLP-1 agonist expression response could not be investigated in pancreatic islets, GLP-1 agonism is known to stimulate brown adipose tissue thermogenesis and browning through hypothalamic AMPK [27]. Thereby, we hypothesized that components of the GLP-1 response beta-cell subnetwork might be functional in both tissues. Our findings provide biological insight into the common mechanisms through which the effects of GLP-1 may be modulated in these tissues.

A few genes from the consensus network were highlighted by additional support from the meta-analysis of the discovery and validation dataset. The four SNPs with the lowest meta-analysis *P*-values had been assigned to the genes *GDI2*, *RYR2*, *KIAA0232*, *COL4A1* and *COL4A2*. Of those, the rs871748 variant (*GDI2*) was in addition found to be nominally associated with four insulin secretion phenotypes in the MAGIC data. The gene product of *GDI2* is a GDP dissociation inhibitor, which is involved in vesicular trafficking between cellular organelles by regulating GDP-GTP exchange reactions of Rab proteins. In the GLP-1 response consensus network, *GDI2* interacts with *RAP1A*, which encodes the Rap1 protein. Rap1 has been shown to be essential for cAMP mediated potentiation of glucose stimulated insulin secretion, such as the one stimulated by GLP-1, through its activation by Epac2[16]. The importance of the Rap1 signaling pathway was further highlighted by it being among the top enriched pathways in the consensus network. Interestingly, the Ryr2 channel encoded by *RYR2* is also activated by Epac2 and plays a role in GLP-1 stimulated insulin secretion by intracellular Ca^2+^ mobilization [15].

The uncharacterized protein *KIAA0232* is another direct interaction partner of *RYR2* in the consensus network and is additionally supported as the causal gene for the associated rs7669558 variant by its strong eQTL association in blood. The rs7669558 SNP is located in a cluster of active islet enhancers, making this protein an intriguing target for further characterization.

The two collagen IV genes, *COL4A1* and *COL4A2*, were supported by the meta-analysis *P*-value for the rs72509 variant and their role in GLP-1 response was additionally supported by evidence from the transcriptome experiment in mice treated with the GLP-1 receptor agonist liraglutide. These two genes are part of the focal adhesion and extracellular matrix-receptor interaction pathways, the two most strongly enriched pathways in the consensus network. The integrin mediated ligation of pancreatic beta-cells to collagen IV is known to promote the secretion of insulin [28] and focal adhesion is important for glucose stimulated insulin secretion [29], which is here highlighted in the context of GLP-1 stimulation. Both of these pathways, in addition to the regulation of actin cytoskeleton pathway, have been implicated in type 2 diabetes, as they were found to be overrepresented among genes differentially methylated in pancreatic islets of type 2 diabetes patients compared to non-diabetic controls [30]. Moreover, the *COL4A1* gene (along with two other consensus network members, *VAV2* and *ITGB5*) was found to be among the significantly hypomethylated genes. In addition, *CD44*, a widely expressed cell surface glycoprotein known to induce integrin-mediated adhesion, was the most significant up-regulator of the expression dataset for liraglutide response [31].

As liraglutide is a GLP-1 receptor agonist, liraglutide treatment-induced gene expression alterations can highlight networks and pathways related to GLP-1 response. In our transcriptomic experiment for liraglutide response in adipose tissue, we observed an overlap with the pathways prioritized by the GLP-1 stimulated insulin secretion consensus network in the pancreatic beta-cell. This suggests that some additional effects of GLP-1 in peripheral tissue could be mediated by genes that are not islet-specific, such as collagen type IV. Studies performed in isolated adipocytes have demonstrated that GLP-1 has the ability to induce both lipogenic and lipolytic mechanisms in white adipose tissue through activation of ERK, PKC and AKT signaling pathways [32]. These are also active in the islets, as transgene expression of the GLP-1R in the islets of *Glp1r*–/–mice restored GLP-1R dependent stimulation of cAMP and Akt phosphorylation and was sufficient for restoration of GLP-1 stimulated insulin secretion in perfused islets [33]. Thereby, the GLP-1 receptors in the islet have an essential physiological role in the regulation of beta-cell function and glucose homeostasis through the Akt pathway, which in parallel affects adipogenesis.

Remodeling in heart and vasculature is linked to alterations in extracellular matrix and integrin expression [34]. This relates to a recently reported clinically relevant downstream effect of GLP-1 stimulation, cardioprotection via the PI3K/Akt/Bad pathway [35], leading to stabilization of atherosclerosis in increase of plaque collagen content in arteriosclerotic mice [36]. Both human and animal model networks presented in the current study point to relevant signals for the PI 3-kinase signaling pathway, which may be important for both insulin secretion and diabetes comorbidities originating in other tissues, such as cardiovascular disease.

One of the main challenges in GWAS is to identify the causal genes mediating the effects of associated variants. Here, each variant was assigned to nearby genes but in addition, the most likely causal gene for each variant can be considered to be the one that also physically interacts with other candidate genes at the protein level in the context of the beta-cell, based on the network module prioritization. We found a number of the consensus network SNPs to either have a direct potential eQTL association with the corresponding gene either in human islets, pancreas or blood, or reside in loci overlapping clustered active islet enhancers that are known to be enriched for type 2 diabetes and fasting glucose associated loci [23], suggesting that many of the SNPs driving the consensus network significance have the potential to confer regulatory effects on gene expression.

A limitation of our study is clearly the limited sample size of the GWAS cohorts impacting statistical power. Nevertheless, the modified hyperglycemic clamp procedure has benefits in terms of producing a very detailed measure, which is more proximal to the phenotype compared to those based on for instance OGTT and thus likely to reveal larger effect sizes, which is a general observation for pharmacogenomic traits [37]. We employed the integrative network analysis to reduce the number of false positives, by prioritizing signals in vicinity of genes that have the potential to physically interact in the pancreatic beta-cell, and furthermore focused our attention on the genetic variants from the network with the best meta-analysis P-values from the two cohorts. The network and transcriptomic analyses provide biological hypotheses in the form of prioritized genes and pathways for future functional studies that will be required to confirm their role in GLP-1 stimulated insulin secretion and as such this study should be seen as an exploratory study.

In conclusion, we have identified a beta-cell PPI network module enriched for nominal associations with GLP-1 stimulated insulin secretion. This network module highlights genes and pathways already known to be of importance for insulin secretion, and indicates new potential target genes that operate in the same network context. The genetic variants prioritized through the network approach were collectively associated with insulin secretion capability in the general population and many overlap with islet-active regulatory regions, suggesting a possible influence on the gene expression of network members. Consistent with this hypothesis, alterations in gene expression in response to liraglutide treatment in mice showed that main network regulators are connected to genes nominally associated with the GLP-1 stimulated insulin response. Furthermore, the results demonstrate how data integration can highlight biological mechanisms underlying a phenotype where GWAS results on their own may be insufficient.

## Materials and methods

### Hyperglycemic clamp cohorts

GLP-1 stimulated insulin secretion was measured with a modified hyperglycemic clamp in 126 twins and sibs from the Netherlands twin register (NTR), aged 20–51 years [2]. This cohort consists of a mixed sample of twins and non-twin sibs recruited from 54 families (family size 2–9). In total, the NTR twin sample included 33 monozygotic twin pairs (n = 66), 14 same sex dizygotic twin pairs (n = 28) and 32 single twins and same sex sibs of the twins. The validation cohort consisted of 100 unrelated subjects, aged 18–68 years, from Tübingen, Germany [10] (68 with NGT/32 with IGT) who were examined with an identical hyperglycemic clamp [38]. The human studies were conducted according to the principles expressed in the Declaration of Helsinki. The medical ethics committee at VU University Amsterdam, the Netherlands, approved the NTR study protocol. The study protocol was approved by the ethical committee of the University of Tübingen, Germany. All participants gave written informed consent before the study was started.

### Hyperglycemic clamp procedure

Hyperglycemic clamp studies were performed in 2005–2007 and 1998–1999 for the NTR and Tübingen cohort respectively. All participants underwent a modified hyperglycemic clamp at 10 mmol/l glucose for three hours with additional GLP-1 and arginine stimulation during the last hour. After a priming infusion of glucose to acutely raise blood glucose levels, blood glucose levels were measured with a glucose analyzer and kept constant at 10 mmol/l during the whole clamp. Insulin levels were measured with immunoassays as previously described. Insulin sensitivity index was calculated as described previously. GLP-1–stimulated insulin release was measured as the mean incremental area under the curve (160–180 min) following GLP-1 stimulation. Exact details of the modified hyperglycemic clamp procedure can be found in Simonis-Bik et al. [2].

### Genotyping and association analysis

Genotyping of the two cohorts (NTR and Tübingen) and subsequent data analysis was performed between September 2013 and September 2016. Genotyping using the HumanCoreExome chip was performed according the manufacturers protocol (Illumina Inc. San Diego, CA, USA). For quality control we used the following settings: a cut-off for the genotyping call rate of 99%, Gentrain and clusters score < 0.6 and 0.4 respectively, and the *P*-value cut-off for Hardy-Weinberg equilibrium was set at 10^−4^. In total 513,444 SNPs passed quality control. Imputation up to the March 2012 1000 Genomes reference panel was done using SHAPEIT (v2.r644) and IMPUTE (v2.3.0). SNPs with a low frequency (MAF < 5%) and or imputation quality (*R*_*T*_^*2*^ < 0.4) were excluded, leaving 6.6 M SNPs for the analysis. The test statistics were not adjusted for inflation (population stratification) because of the low genomic inflation factor (λ = 1.02). In order to account for the family relationships in the twin cohort we used QTassoc [39], a software tool based on SNPtest that is capable of handling familial data (using the kinship coefficients matrix) and genotype uncertainty. Data from both cohorts were analyzed using linear regression under an additive model and were adjusted for age, gender, glucose tolerance status, insulin sensitivity index, and familiarity (NTR only) as potential confounders. Fixed-effect meta-analysis (quantitative trait) of the two studies was performed using GWAMA [40] with double genomic control, i.e. for the results from the individual studies (-gc) and from the meta-analysis (-gco).

### Gene significance scoring

*P*-values from the discovery GWAS analysis on GLP-1 stimulated insulin secretion were used to assign gene significance. Each gene was assigned the lowest *P*-value mapping to its boundaries, defined as 110kb upstream and 40kb downstream from transcription start/stop sites. These boundaries represent the 99^th^ percentile of cis-eQTLs from their associated genes [41]. All SNPs not mapping to any predefined gene-window were excluded from the analysis.

### Construction of a beta-cell specific PPI network

A beta-cell specific PPI network was created by pruning the InWeb database [42] of high confidence physical PPIs (154,168 interactions between 12,778 proteins) using published beta-cell specific RNAseq data [13]. More specifically, genes with 25th percentile RPKM < 1 were considered less likely to be expressed in the beta-cell and thus removed from the pruned beta-cell specific network. Special care was taken not to remove beta-cell or pancreatic transcription factors (S9 Table) or other lowly expressed beta-cell specific genes, such as the ones defined as beta-cell enriched in the study by Nica et al. [13].

### Identifying hotspots in the PPI network

The Cytoscape plugin jActiveModules [14] was used to identify modules in the network that were enriched for high scoring genes (that is with low SNP *P*-values). The jActiveModules algorithm is described in detail in the original publication [14] but in brief, each node representing a gene *i* in the network is assigned a z-score *z*_*i*_ = Φ^−1^(1 − *p*_*i*_), where Φ^−1^ is the inverse normal cumulative distribution function and *p*_*i*_ is the gene significance score. An aggregated z-score is calculated for each module *A* with *k* nodes as the normalized sum of z-scores of all genes in the module:

$$z_{A}=\sum_{i\in A}Z_{i}/\sqrt{k}$$

A module z-score > 3 is generally considered significant, according to the jActiveModules authors. jActiveModules searches for top scoring modules within the full network, starting from each of the (in our case top 100 highest scoring) nodes in the beta-cell network and adding nodes to the network module using a greedy search algorithm.

The z-scores generated by jActiveModules are a measure of the enrichment significance of the modules compared to 100,000 permutations of randomly selected genes from the whole beta-cell PPI network. The z-scores were additionally compared to 10 sets of permuted gene significance values. As the top scoring module was too large (n = 179 genes) for manual inspection, the jActiveModules algorithm was rerun on the top scoring module and the consequent submodules within it used to build a consensus network, where a node was included if it appeared more than once in any of the top 15 second order modules. The enrichment of association signals in the consensus network was validated in the Tübingen cohort by comparing the combined z-score for the network SNPs to those obtained from 100,000 randomly sampled sets of SNPs that had previously been assigned to each of the genes in the beta-cell network during the gene significance scoring step. For comparison we attempted the same validation for a matched number of the top independent signals from the discovery GWAS.

### Gene set enrichment of beta-cell specific PPI network module

Gene set enrichment analysis was performed using ConsensusPathDB [43], testing Gene Ontology (GO) and KEGG pathway gene sets and using all 8,457 genes present in the beta-cell PPI as background. Benjamini & Hochberg [44] adjusted *P*-values < 0.05 were considered statistically significant.

### eQTL associations

We extracted eQTL associations for each of the SNP-gene pairs in the consensus network from the GTEx portal [45] and from human pancreatic islets [46]. We searched for eQTL associations in human islets (n_samples_ = 118), pancreatic tissue (n_samples_ = 58) and blood (n_samples_ = 168). Due to the predefined SNP-gene pairs tested and the small number of samples, we considered SNPs with *P* < 0.01 as potential eQTLs. In addition we looked up the network SNPs amongst significant eQTLs in whole blood from 5,311 individuals [47].

### Islet regulatory element overlap

Positions for six types of regulatory elements in pancreatic islets (promoters, inactive enhancers, active enhancers, clustered active enhancers, CTCF bound sites and other) were obtained from a recent study [23]. All SNPs in the consensus network and SNPs in high LD (r^2^ > 0.8) were tested for overlap with any of the regulatory regions.

### Polygenic risk scores

Weighted PRS were tested for association with oral glucose tolerance test phenotypes (S4 Table) from MAGIC [11]. The association testing was performed with the “gtx” R package. The analysis was limited to SNPs common to both datasets. The effect raising allele of the best SNP for each gene (based on its discovery GWAS *P*-value) was chosen for the PRS, and weighted by the effect size in the discovery GWAS. For comparison, another PRS was created from a matched number of the top discovery GWAS SNPs (LD-pruned at r^2^ < 0.8).

### Liraglutide treatment in ob/ob mice

Twenty female B6.Cg-Lep ob/ob mice were obtained from Charles River (Sulzfeld, Germany) in an age of 6–8 weeks. Upon receipt, animals were housed 5 per cage in an air-conditioned, pathogen-free barrier facility maintained at 22±2°C with an air humidity of 45–65% and a 12-h dark–light cycle (lights on at 06:00 am). The mice had ad libitum access to a standard rodent diet from ssniff® (R/M-H, V1534-0, 10 mm pellets, Soest, Germany) and tap water during the entire experiment. The mice were surgically implanted in an age of 10–12 weeks with Alzet osmotic mini pumps (model 1002, 0,25μl/h, Cupertino, CA, USA) under isoflurane (2.5% using a flow of 0.6 L/min) inhalation anesthesia with peri-operative, subcutaneous, analgesic Buprenorphine treatment (0.05 mg/kg Temgesic®). Due to wound healing problems after the mini pump surgery three mice were sacrificed during the time course of the experiment and therefore are not represented in the study data. The mice were split into two groups to perform continuous subcutaneous infusions of Dulbecco's phosphate buffered saline (vehicle; n = 8) or GLP-1 (GLP-1; n = 9) agonist Liraglutide (Victoza®, batch# CS6C214, Novo Nordisk A/S, Bagsvaerd, Denmark) to investigate the effects of the respective treatment on peri-gonadal white adipose tissue gene expression. Vehicle or Liraglutide at a dose of 600 μg/kg/d were continuously infused (at a dose of 600 μg/kg/d) over a 14 days period. On the morning after the osmotic mini pump reservoirs liquid content should have been consumed mice were dissected to remove the peri-gonadal fat pad for white adipose tissue gene expression analysis. To dissect the white adipose tissue, mice were anaesthetized under isoflurane (3.0% using a flow of 0.6 L/min) inhalation anesthesia and finally sacrificed by cervical dislocation. At least 220 mg of white adipose tissue was quickly removed, shock-frozen in liquid nitrogen and thereafter stored at -80°C for mRNA extraction. The animal study conformed to the German law for the protection of animal guidelines and the guide for the care and use of laboratory animals published by the US National Institutes of Health (NIH Publications No 85–23, revised 2011) as well as to Sanofi-Aventis Ethical Committee guidelines and were approved by a local authority ethics review board (RP Darmstadt).

### Transcriptome profiling of liraglutide treated mice and baseline controls

To perform RNA-Seq analysis, samples were single-end sequenced at a depth of 75 – 80M reads per sample with a read-length of 51 bp using an Illumina Hiseq2500. Raw sequencing files were quality controlled with FastQC [48]. Alignment and trimming of reads was performed using the OSA [49] algorithm against the mouse reference genome b38.1 with RefSeq as the gene model as implemented in OmicSoft® ArraySuite® software, version 8. RNA transcripts were quantified using RSEM methods [50] as implemented in Arraystudio counting count the read fragments mapping to each individual gene and quantify expression by the corresponding FPKM. In summary, expression was measured as FPKM for 25,054 unique genes. Principal component analysis was then performed to check for possible batch effects and outliers complemented by calculating the RNA-Seq 5'->3' trend for each sample. One sample for the vehicle control as well as for the Liraglutide group were identified as outliers and removed from subsequent analysis, resulting in 7 and 8 samples remaining for each group respectively. Abundance values (counts) were normalized and compared between liraglutide treated mice versus baseline controls using DESeq2 [51]. All *P*-values were adjusted for multiple testing by the Benjamini-Hochberg method [44].

### Gene set enrichment analysis of the transcriptome profiling of liraglutide treated mice vs. baseline controls

Gene set enrichment analysis was performed using QIAGEN's Ingenuity® Pathway Analysis, a web-based bioinformatics tool [Qiagen, Redwood City, CA, USA]. A given set of input genes was associated with molecular networks based on their connectivity in the Ingenuity Pathways Knowledge Base. Fisher’s exact test was used to determine the probability that each biological function assigned to that data set was attributable to chance alone. The goal of the IPA Upstream Regulator analytic is to identify the cascade of upstream transcriptional regulators (any molecule that can affect the expression of other molecule) that can explain the observed gene expression changes in a given dataset. For each potential transcriptional regulator two statistical measures, an overlap *P*-value and an activation z-score are computed. The overlap *P*-value calls likely upstream regulators based on significant overlap between dataset genes and known targets regulated by a transcriptional regulator. The activation z-score is used to infer likely activation states of upstream regulators based on comparison with a model that assigns random regulation directions (S8 Table). Analyses included direct and indirect relationships that have been experimentally observed in mice, rat or human studies. All differentially expressed genes (n = 342 with a FDR < 0.05) were used for the analysis.

### Tissue specific network analysis of genes identified in both human and animal experiments

To verify if the genes identified in the independent human and mouse experiments had interactions in specific interaction networks for the islet and adipose tissue, we used the Genome-scale Integrated Analysis of gene Networks in Tissues–GIANT [25]. GIANT leverages a tissue-specific gold standard to automatically up-weight datasets relevant to a tissue from a large data compendium of diverse tissues and cell-types. The resulting functional networks accurately capture tissue-specific functional interactions. We only included interactions with a relationship confidence > 0.28 (within the range of 0 to 1).

## Supporting information

S1 Fig Q-Q plot of the GWAS on GLP-1 stimulated insulin secretion as measured with the hyperglycemic clamp in the NTR cohort. (TIF)

S2 Fig Manhattan plot of the GWAS on GLP-1 stimulated insulin secretion as measured with the hyperglycemic clamp in the NTR cohort. (TIF)

S3 Fig Flowchart illustrating the analysis workflow. **A** The discovery analysis consisted of an integrative network analysis where GLP-1 stimulated insulin secretion GWAS P-values were combined with a beta-cell specific PPI network to identify enriched network modules or ‘network hotspots’. The top scoring network module was distilled into a smaller consensus network, by combining top selected nodes from a second network module search. **B** Functional annotation of the consensus network genes and SNPs consisted of pathway overrepresentation analysis, eQTL lookups in pancreatic islets and blood and overlaps with islet regulatory elements (promoters and enhancer clusters). **C** We attempted validation of the network by calculating a combined z-score for the top-scoring network SNPs in an independent cohort and investigating polygenic risk scores from the same SNPs in OGTT data from MAGIC investigators. These results for the network SNPs were compared to those obtained by a matched number of top GWAS SNPs. Finally the results from a study of the effects of liraglutide (a GLP-1 agonist) on mouse adipose tissue were compared to the findings from the network and pathway analysis of human genomic data.
(TIF)

S4 Fig Network module z-scores derived from real (red) and randomized (black) gene significance scores. Network module z-scores based on randomized gene significance scores are shown as the mean of 10 randomizations with 95% confidence intervals (SEM*1.96).
(TIF)

S5 Fig The GO terms enriched (BH adjusted *P*-value < 1 × 10^−3^) within the GLP-1 response network, compared to the whole beta-cell PPI network. Level 2 GO terms are shown for each of the categories; molecular function (red), cellular component (yellow) and biological process (blue).
(TIF)

S6 Fig The percentage of GLP-1 stimulated insulin secretion consensus network loci (green) overlapping islet regulatory elements, shown in comparison to genome-wide significant fasting glucose associated loci (blue), genome-wide significant T2D associated loci (red) and loci with *P* < 1 × 10^−4^ in a GWAS of corrected insulin response adjusted for insulin sensitivity index (orange). (TIF)

S7 Fig The combined z-score in the Tübingen validation cohort for the top 31 independent GWAS SNPs (red line) compared to 100,000 z-scores obtained from randomly selected sets of SNPs from the beta-cell network (histogram), empirical *P*-value = 0.95. (TIF)

S8 Fig Mouse experiment overview. B6.Cg-Lep ob/ob mice were treated with 600 μg/kg/d liraglutide (n = 9) or vehicle (n = 8).
(TIF)

S9 Fig Visualization of subnetworks prioritized from the mouse adipose tissue transcriptome experiment. A) Collagen genes interact with genes that also appear in the human consensus network, and which connect the collagen pathway to the GLP-1 receptor. Gene nodes are colored by up- (red) and down- (green) regulation in the liraglutide treated animals versus untreated controls. B) *CD44* is an upstream regulator of collagen genes and *CTNNB1*. Interactions are based on the Ingenuity Pathway Analyses library.
(TIF)

S10 Fig Tissue-specific functional interaction networks from GIANT. Relevant genes (largest nodes) overlapping in the human and mouse studies were used to query tissue-specific interactions. The networks indicate that these genes have functional interactions in both pancreatic islet (A) and adipose tissue (B). Minimum relationship confidence is highlighted by the colors from green (0) to dark red (1).
(TIF)

S1 Table The six independent signals from the discovery GWAS on GLP-1 stimulated insulin secretion with *P* < 1 × 10^−5^, well as the validation and meta-analysis statistics. (XLSX)

S2 Table An overview of the genes in the GLP-1 response consensus network. The SNP with the minimum discovery GWAS *P*-value mapping to each gene is shown together with the discovery GWAS, validation and meta-analysis statistics.
(XLSX)

S3 Table An overview of the eQTL and islet regulatory element overlap lookup for the GLP-1 response consensus network SNPs. (XLSX)

S4 Table OGTT-derived phenotypes for which summary statistics from MAGIC were used to investigate SNP and polygenic risk score associations. Table adapted from Prokopenko et al. [11]
(XLSX)

S5 Table Nominally significant (*P* < 0.05, highlighted in bold) associations between SNPs from the GLP-1 response consensus network and quantitative metabolic traits from MAGIC. The last column shows the effect (beta) of the effect allele used in MAGIC on GLP-1 stimulated insulin secretion in the NTR cohort.
(XLSX)

S6 Table The association between weighted polygenic risk scores for SNPs in the GLP-1 response consensus network with discovery GWAS *P* < 5 × 10^−4^ and OGTT-derived phenotypes from MAGIC. *P*-values < 0.05 are highlighted in bold. The phenotypes are described in more detail in S4 Table.
(XLSX)

S7 Table The association between weighted polygenic risk scores for LD-pruned top GWAS SNPs (matched number of SNPs compared to S6 Table) and OGTT-derived phenotypes from MAGIC. (XLSX)

S8 Table Upstream regulators identified in the transcriptome experiment of differentially expressed genes in liraglutide treated ob/ob mice versus baseline controls. The overlap *P*-value calls likely upstream regulators based on significant overlap between dataset genes and known targets regulated by a transcriptional regulator. The z-score algorithm is designed to produce either a prediction of activation or inhibition (or no prediction). The analysis examines the known targets of each upstream regulator in the dataset, compares the targets’ actual direction of change to expectations derived from the literature, then issues a prediction for each upstream regulator. The direction of change is the gene expression in the experimental samples relative to a control. If the direction of change is consistent with the literature across most targets, IPA predicts that the upstream regulator is more active in the experimental sample than in the control. Mostly inconsistent with the literature (anti-correlated with the literature), IPA predicts that the upstream regulator is less active in the experimental sample than in the control. If there is a random pattern relative to the literature, IPA does not make an activation or inhibition prediction for the upstream regulator. However, in these case, there may still be a significant overlap (Fisher’s Exact *P*-value), just no clear pattern to predict a direction of activation. Genes highlighted in yellow were also present in the human beta-cell GLP-1 response consensus network.
(XLSX)

S9 Table Islet and pancreatic transcription factors that were specifically included in the beta-cell PPI network. (XLSX)
