# Supplementary figures and images for: Integrative network analysis highlights biological processes underlying GLP-1 stimulated insulin secretion: A DIRECT study
Source: PLoS One. 2018 Jan 2;13(1):e0189886. doi: 10.1371/journal.pone.0189886 (PMC5749727; doi:10.1371/journal.pone.0189886)

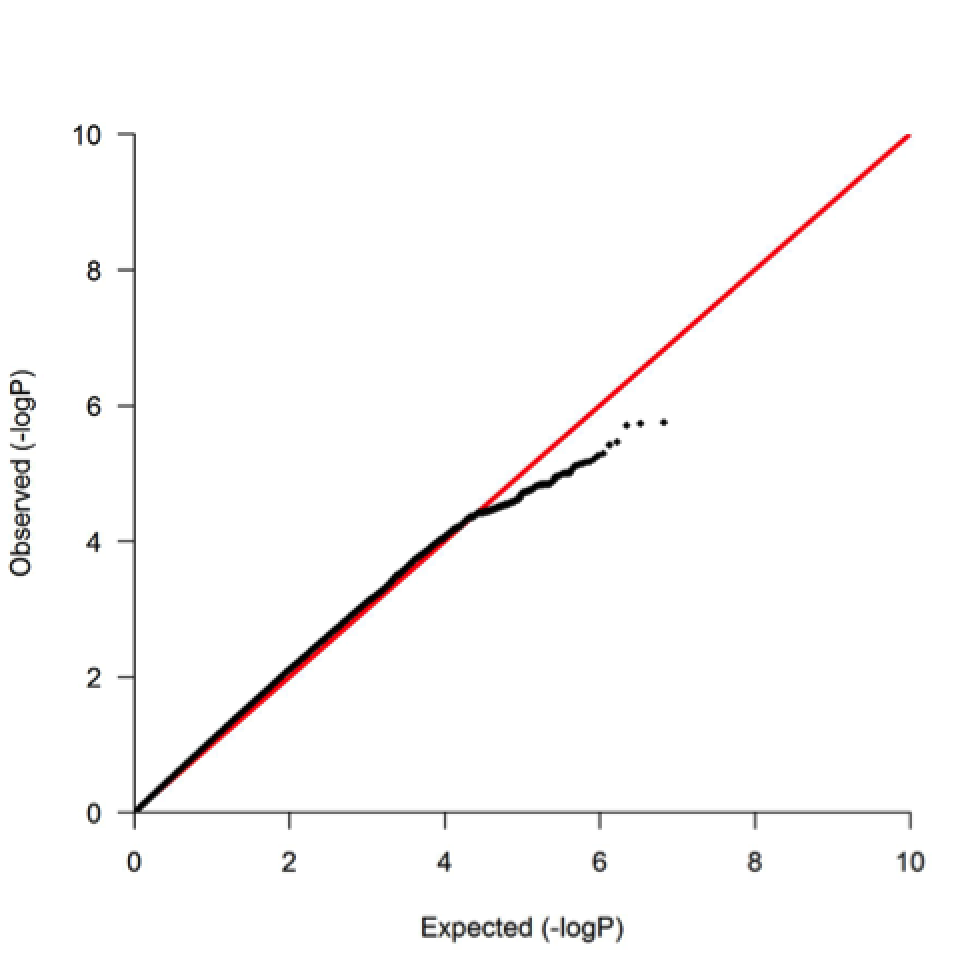

Supplement: S1 Fig — (TIF) [file pone.0189886.s001.tif]

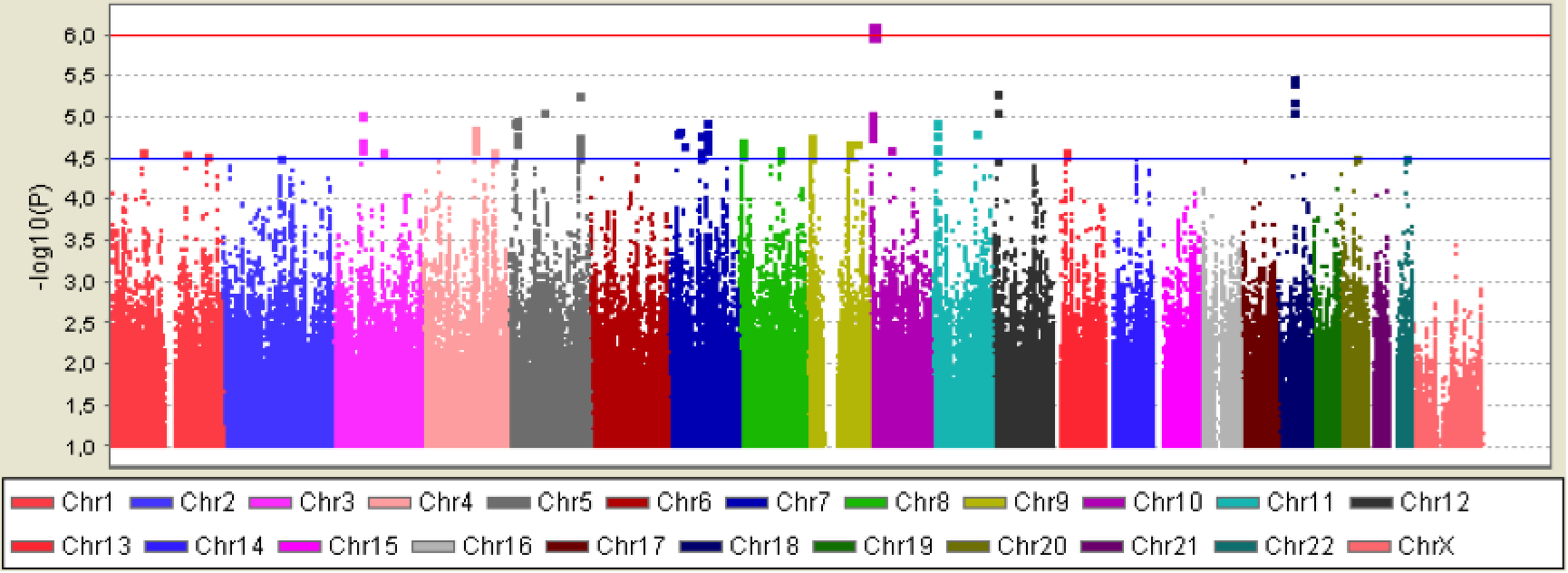

Supplement: S2 Fig — (TIF) [file pone.0189886.s002.tif]

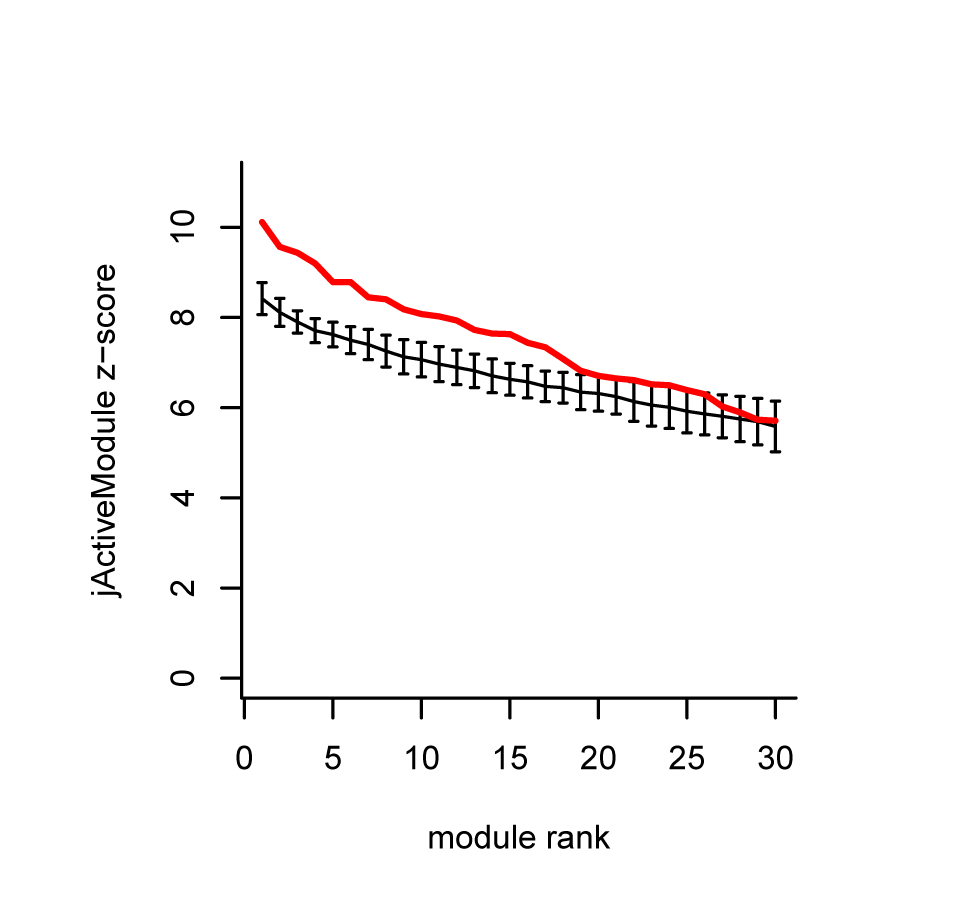

Supplement: S3 Fig — A The discovery analysis consisted of an integrative network analysis where GLP-1 stimulated insulin secretion GWAS P-values were combined with a beta-cell specific PPI network to identify enriched network modules or ‘network hotspots’. The top scoring network module was distilled into a smaller consensus network, by combining top selected nodes from a second network module search. B Functional annotation of the consensus network genes and SNPs consisted of pathway overrepresentation analysis, eQTL lookups in pancreatic islets and blood and overlaps with islet regulatory elements (promoters and enhancer clusters). C We attempted validation of the network by calculating a combined z-score for the top-scoring network SNPs in an independent cohort and investigating polygenic risk scores from the same SNPs in OGTT data from MAGIC investigators. These results for the network SNPs were compared to those obtained by a matched number of top GWAS SNPs. Finally the results from a study of the effects of liraglutide (a GLP-1 agonist) on mouse adipose tissue were compared to the findings from the network and pathway analysis of human genomic data. (TIF) [file pone.0189886.s003.tif]

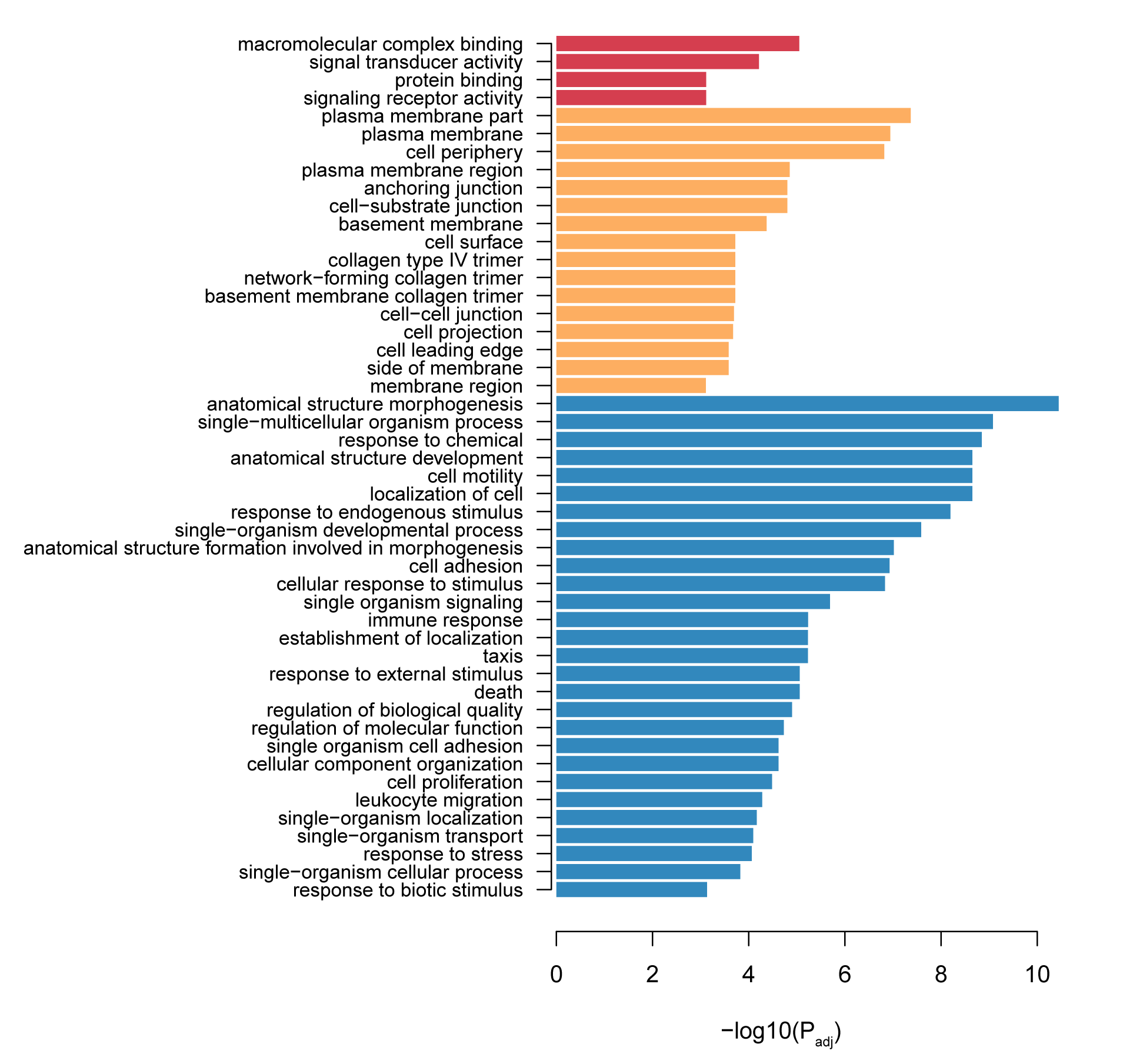

Supplement: S4 Fig — Network module z-scores based on randomized gene significance scores are shown as the mean of 10 randomizations with 95% confidence intervals (SEM*1.96). (TIF) [file pone.0189886.s004.tif]

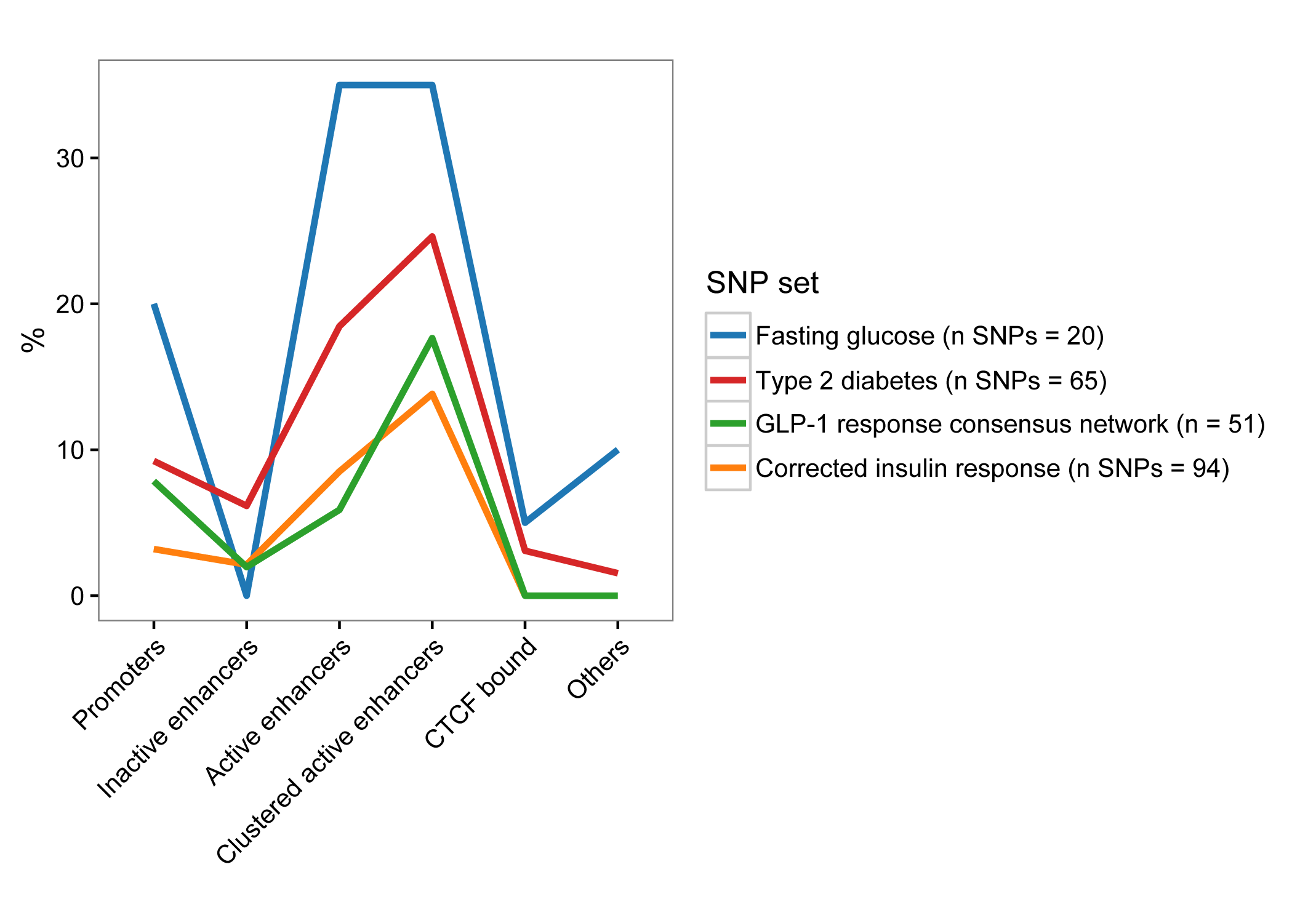

Supplement: S5 Fig — Level 2 GO terms are shown for each of the categories; molecular function (red), cellular component (yellow) and biological process (blue). (TIF) [file pone.0189886.s005.tif]

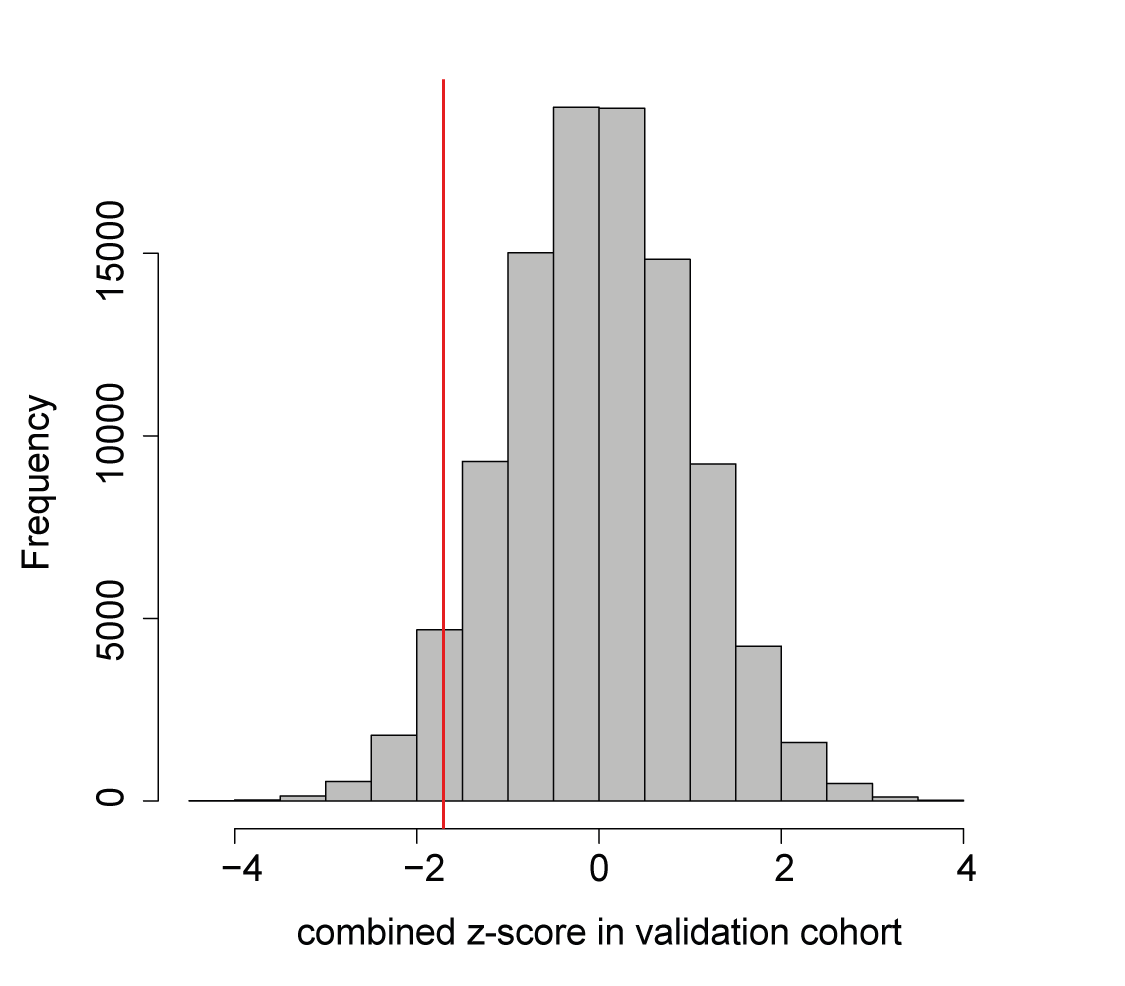

Supplement: S6 Fig — (TIF) [file pone.0189886.s006.tif]

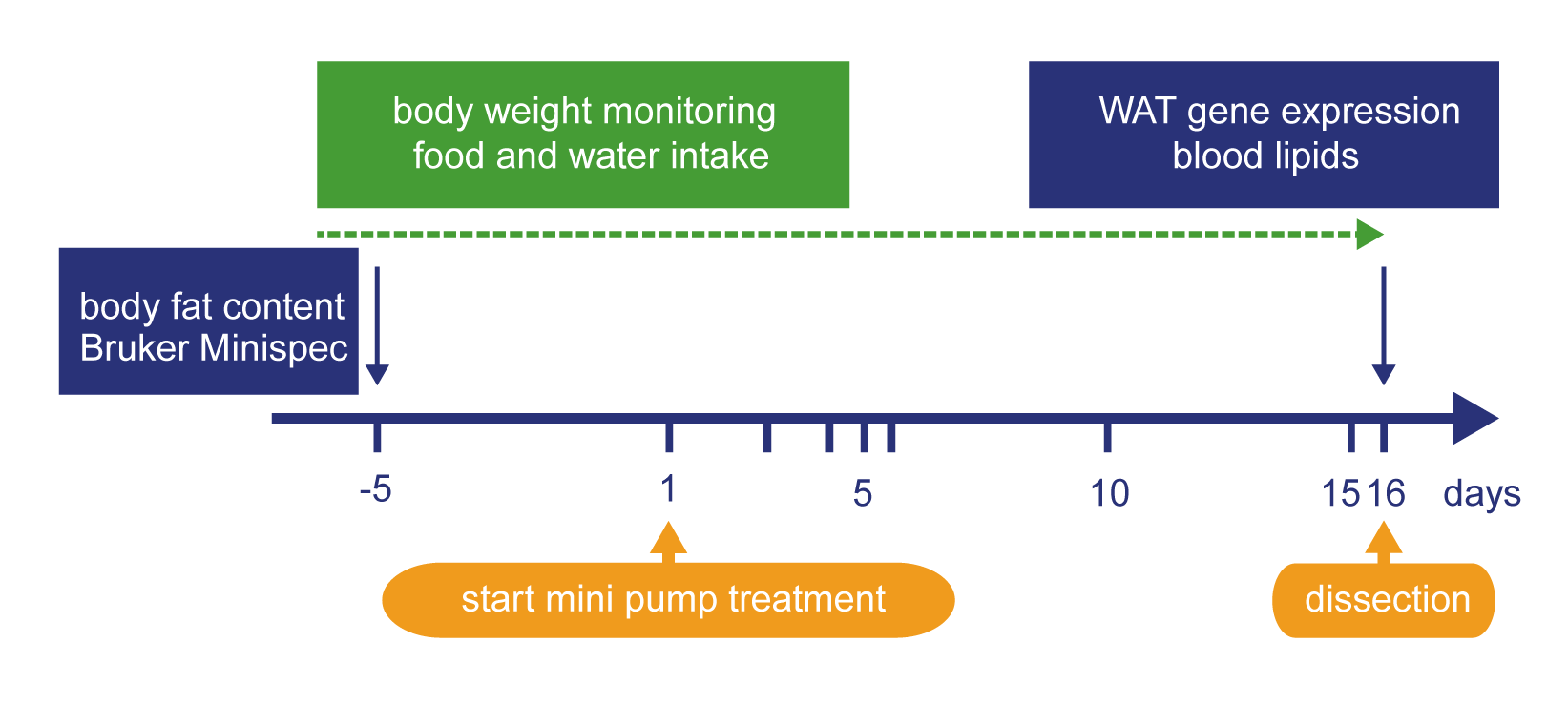

Supplement: S7 Fig — (TIF) [file pone.0189886.s007.tif]

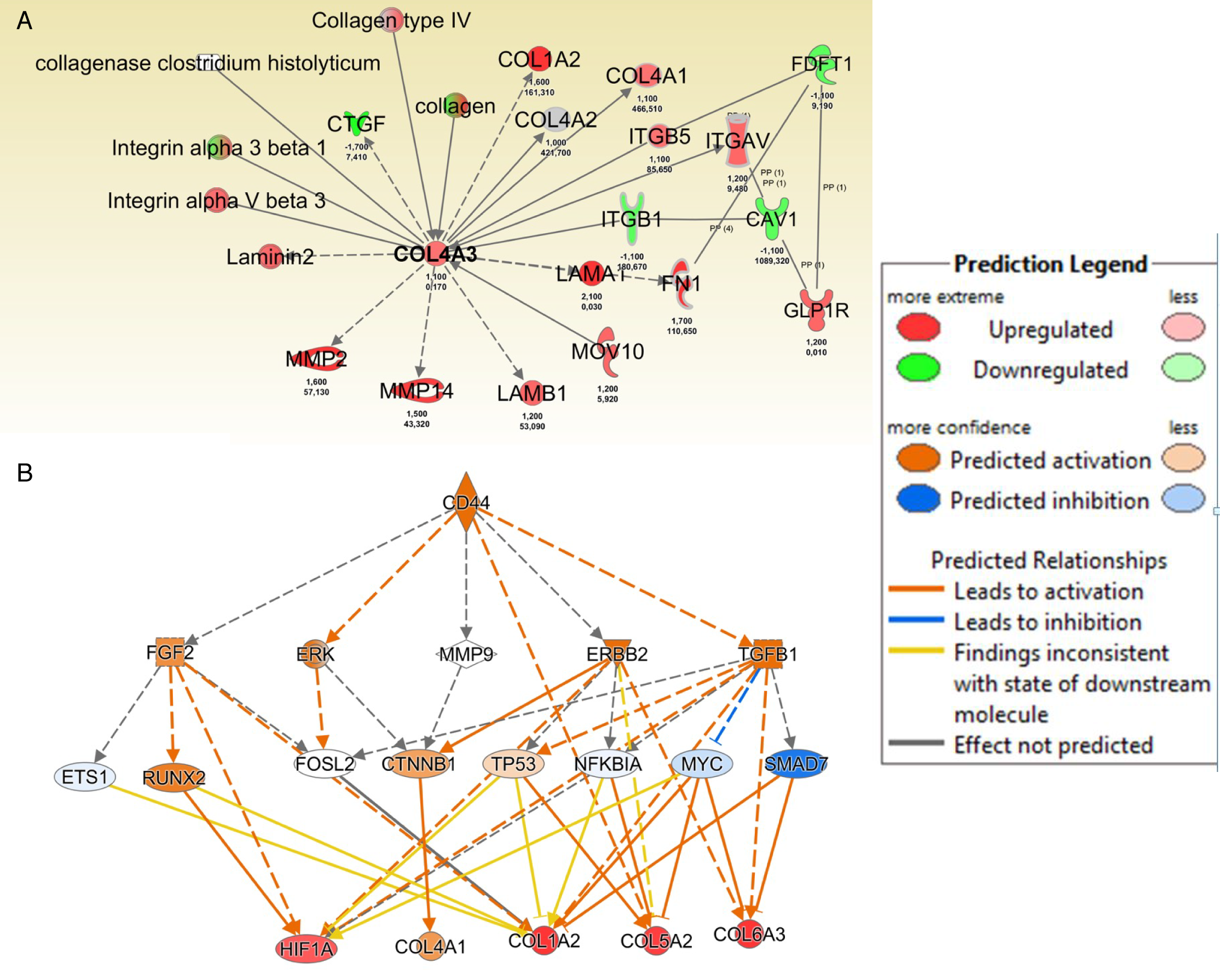

Supplement: S8 Fig — B6.Cg-Lep ob/ob mice were treated with 600 μg/kg/d liraglutide (n = 9) or vehicle (n = 8). (TIF) [file pone.0189886.s008.tif]

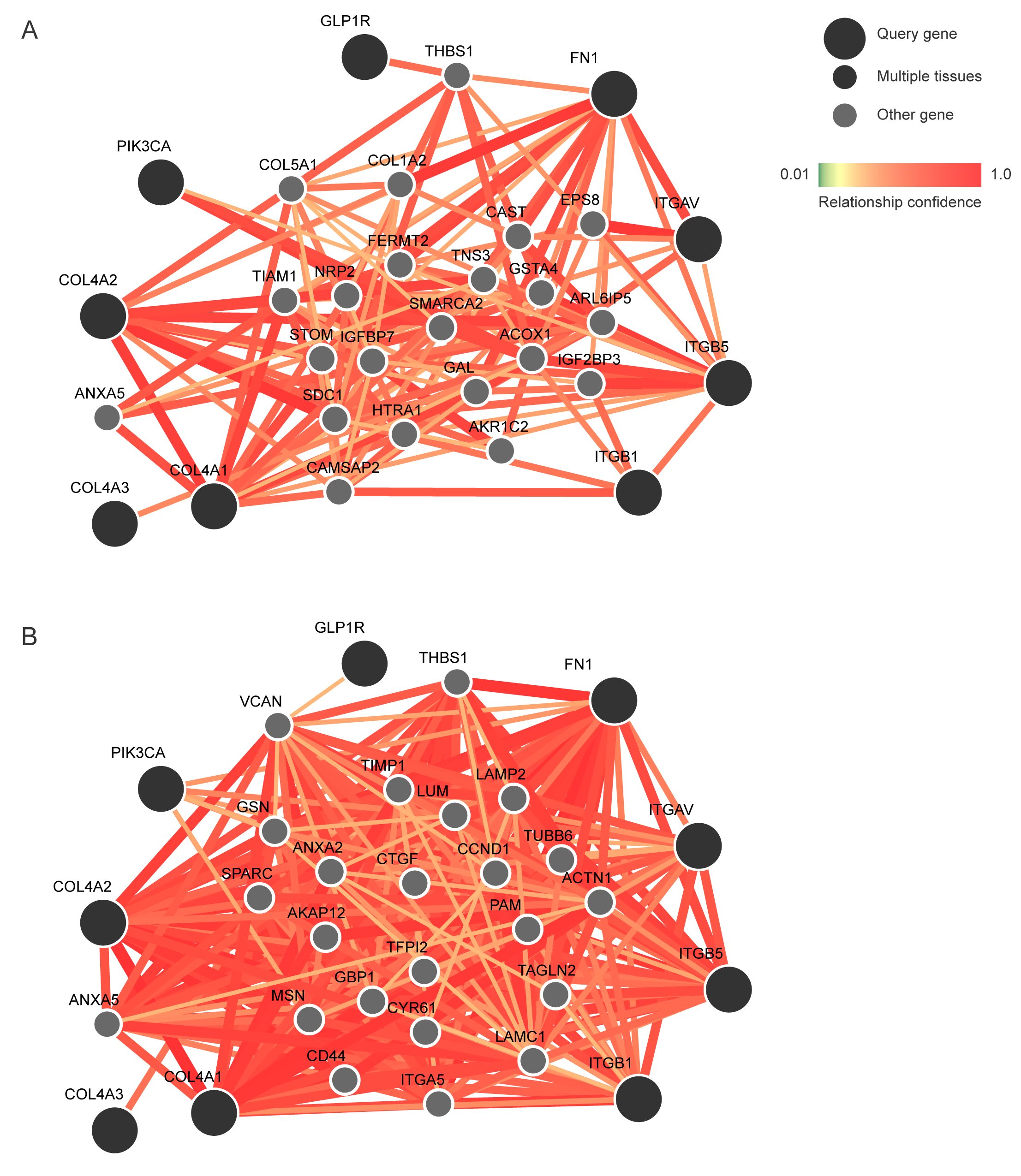

Supplement: S9 Fig — A) Collagen genes interact with genes that also appear in the human consensus network, and which connect the collagen pathway to the GLP-1 receptor. Gene nodes are colored by up- (red) and down- (green) regulation in the liraglutide treated animals versus untreated controls. B) CD44 is an upstream regulator of collagen genes and CTNNB1. Interactions are based on the Ingenuity Pathway Analyses library. (TIF) [file pone.0189886.s009.tif]
